# Does Toothache Before a Dental Visit Associate with the Risk of a First Myocardial Infarction?

**DOI:** 10.3390/jcm14196729

**Published:** 2025-09-24

**Authors:** Dan Sebring, Kåre Buhlin, Thomas Kvist

**Affiliations:** 1 Department of Endodontology, Institute of Odontology, Sahlgrenska Academy, University of Gothenburg, 40530 Gothenburg, Sweden; 2 Division of Periodontology, Department of Dental Medicine, Karolinska Institutet, 14152 Huddinge, Sweden; 3 Department of Oral and Maxillofacial Diseases, University of Helsinki, FI-00014 Helsinki, Finland

**Keywords:** ischemic heart disease, myocardial infarction, toothache, orofacial pain, endodontics

## Abstract

**Background/Objectives**: Ischemic heart disease is typically characterized by chest pain that sometimes radiate to other areas, including the orofacial region. Atypical clinical presentation of cardiac disease risks leading to a delay in the diagnosis and treatment. Symptoms in the orofacial region may also lead to unnecessary dental interventions. The objective of this study was to assess occurrence of toothache, or other oral problems, that prompted a visit to a dental office prior to a first myocardial infarction. **Methods**: In 2010 until 2014, a total of 805 patients hospitalized for a first myocardial infarction and 805 controls matched for age, sex, and postal code area, were recruited to the case–control study PAROKRANK (Periodontitis and its relation to cardiovascular disease). In addition to medical and oral examinations that included panoramic radiography and blood sampling, all participants responded to a survey that covered questions related to oral habits and dental service use. The present study focused on responses to questions concerning the most recent visit to a dental office, specifically if toothache, chewing problem, and/or other problems with the teeth were present, whilst also taking endodontic variables into consideration. **Results**: Time since the most recent visit to a dental office ranged between 0–14 years, with a mean value of 1.08 years and no difference between patients and controls. A majority of responders (80.9%) gave the reason to be a routine dental examination. Toothache as the reason was reported by 146 (11.5%) respondents: 71 (10.9%) patients and 75 (12.1%) controls (*p* = 0.59). No difference was observed between patients and controls. **Conclusions**: Within the limitations of the present study design, seeking dental care for toothache was not associated with the risk of a subsequent first myocardial infarction.

## 1. Introduction

Cardiovascular disease, and ischemic heart disease (IHD) in particular, remains the leading cause of death in adults globally, responsible for almost 9 million deaths in 2021 [1]. IHD results from buildup of atherosclerotic plaques of the coronary arteries. Stiffening and narrowing of the blood vessel reduce blood flow and in case of plaque rupture, formation of a thrombus risks causing total occlusion, ischemia and necrosis of the heart muscle. Clinical manifestations of IHD include angina pectoris and acute myocardial infarction (MI).

Inflammation has been recognized as an important factor in the progression of atherosclerosis and the development of IHD [2]. Successive scientific findings in the last few decades give credence to a relationship between oral inflammatory diseases and IHD [3]. Endodontic inflammatory disease, i.e., pulpitis and apical periodontitis, are localized inflammatory conditions in response to a bacterial infection with high activity of innate and adaptive immune cells, as well as local release of various inflammatory cytokines [4]. Pulpitis and apical periodontitis may be severely painful but can be successfully treated by means of endodontic treatment, such as pulpotomy and root canal treatment.

Whilst the majority of painful conditions in the orofacial area are of dental, periodontal, sinus, or musculoskeletal origin, oral pain may also originate from other areas; so-called heterotopic pain. The cardinal symptoms of IHD are chest pain that is typically provoked by physical activity or stress. However, convergence of afferent nerve fibers may result in pain that radiates to other areas, including shoulders, scapulas, neck, and jaws. Some IHD patients may even experience pain exclusively in other areas than the chest [5]. Unfortunately, a lack of chest pain is one of the most important causes for missed diagnosis of acute MI [6]. When presented in the orofacial region, heterotopic pain of cardiac origin may lead to unnecessary dental procedures and a delay in the diagnosis and treatment of the cardiac disease.

PAROKRANK (periodontitis and its relation to cardiovascular disease) is a case–control study on 805 MI patients and 805 matched controls. In 2016, Rydén et al. reported that periodontal disease is associated with an increased risk of a first MI [7]. Likewise, endodontic disease, including dental caries and untreated apical periodontitis, was associated with the risk of MI in a subgroup of younger individuals [8]. The aim of the present study was to assess, among MI patients and their matched controls, the occurrence of toothache that prompted a visit to a dental office prior to a first MI and enrollment in the PAROKRANK study.

## 2. Materials and Methods

### 2.1. Study Population

**Figure 1:**
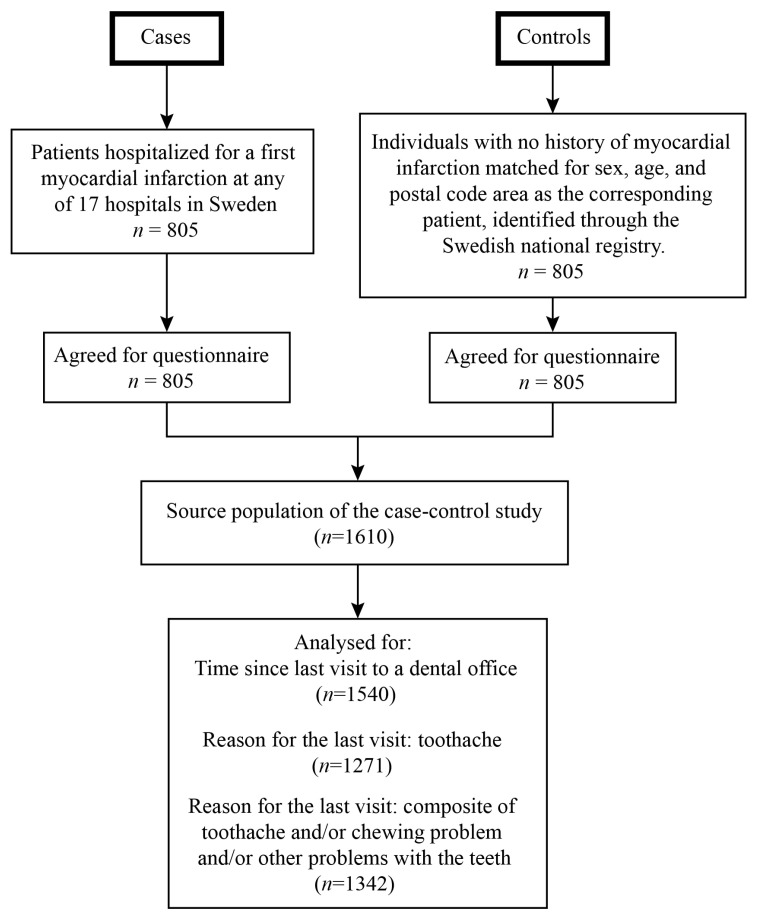
Flow chart of the study.

The PAROKRANK case–control study has been presented previously [7]. In 2010 until 2014, a total of 805 patients hospitalized for a first MI were recruited from 17 Swedish hospitals. Diagnosis of MI was achieved according to international criteria as issued during the study period [9]. Controls matched for sex, age (±3 months), and postal code area were randomly selected from the national population registry. To be eligible for inclusion, controls were to be free from previous MI and heart valve replacement. At the local departments of cardiology and hospital dentistry, patients were scheduled for outpatient visits approximately 6–10 weeks after the acute event, and controls were contacted within ten days. Physical examination and blood sampling was performed, and medical and family history information was collected using standardized questionnaires. A survey comprising a wide range of questions, among them some that concerned oral habits and dental service use, was responded to by all participants. A flow chart of the study is presented in Figure 1.

### 2.2. Assessment of Endodontic Variables

All participants underwent an oral examination that included taking a panoramic radiograph. Endodontic variables, including number of root filled teeth and number of teeth with periapical lesions, were assessed by three calibrated observers using the image software ImageJ (Image Tool 3.0 software program, Department of Dental Diagnostics Science, University of Texas Health Science Center, Houston, TX, USA). Root filled teeth were defined as teeth with radiopaque material in the pulp chamber and/or root canals. Teeth with periapical lesions were defined as teeth with a visible apical or periapical radiolucency that was noticeably larger than the width of the periodontal ligament. In order to reduce false-positive recordings, all observers were encouraged to record disease only when confident of its presence. Agreement with a reference standard ranged between weighted Cohen Kappa 0.98–0.99 for assessment of root filled teeth and 0.54–0.75 for assessment of teeth with periapical lesions [10].

### 2.3. Survey Description and Assessment of Test Variables

The survey was written in Swedish and administered directly to the participants in conjunction with the physical examination. In total, the survey comprised 131 questions that concerned background information, such as marital status, education and occupation, and questions related to systemic diseases, medication, tobacco use, eating habits, physical activity, perceived quality of life and more. A total of 36 questions were allocated to oral health and related topics. The majority of questions were close-ended questions for which the respondent had a choice of answers. A few questions were open-ended and allowed for additional comments.

The present study focuses on questions concerning the most recent visit to a dental office prior to the MI event (patients) or inclusion in the PAROKRANK study (controls). The respondents were asked during which year their most recent visit to a dental office had taken place. Time since the last visit to a dental office was calculated by subtracting the response year from the year of inclusion to the PAROKRANK study.

The reason for the last visit to a dental office was determined by close-ended questions with predefined alternatives, including routine examination, toothache, chewing problem, other problems with the teeth, or improving the appearance of the teeth. Respondents were able to respond “yes” or “no” to multiple alternatives. In the analysis, toothache and a composite variable were tested as predictors of a first MI. The composite variable was defined as a “yes” response to toothache and/or chewing problem and/or other problems with the teeth as the reason for the last visit to a dental office.

### 2.4. Statistical Analysis

Statistical analysis was performed using SAS system 9.4 (SAS Institute, Cary, NC, USA). For comparison between the groups, Fisher’s Exact test (lowest 1-sided value multiplied by 2) was used for dichotomous variables, the Mantel–Haenszel Chi-square test was used for ordered categorical variables, the Chi-square test was used for non-ordered categorical variables and Fisher’s non-parametric test was used for continuous variables. The main results are presented as unadjusted odds ratios (OR) with a 95% confidence interval (CI), together with OR (95% CI) adjusted for age, sex, time since last visit to a dental office, and endodontic variables (number of teeth with periapical lesions, number of root filled teeth, and number of root filled teeth with periapical lesions).

## 3. Results

### 3.1. PAROKRANK Study Subject Characteristics and Survey Responses

**Figure 2:**
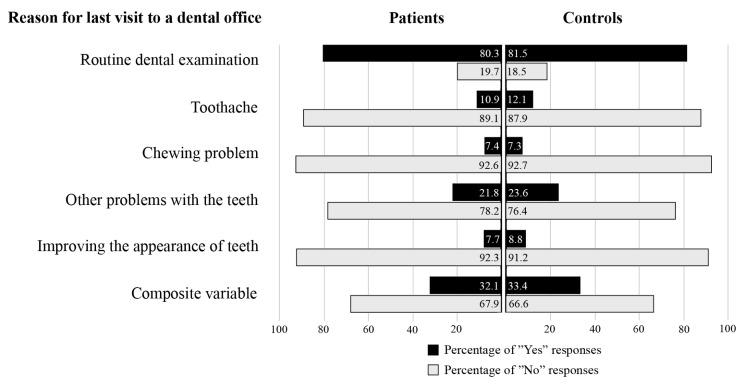
The alternatives for reason for the last visit to a dental office and the responses given by the patients and the controls, respectively. Composite variable is a “Yes” response to toothache and/or chewing problem and/or other problems with the teeth.

Mean age of the participants of the PAROKRANK study was 62 ± 8 years, and the majority (81.2%) were men. Assessment of time since the last visit to a dental office was possible for 772 (95.9%) patients and 768 (95.4%) controls. Table 1 presents time since last visit to a dental office, and the reason for said visit. Time since last visit to a dental office ranged between 0 and 14 years, with no difference between patients and controls (mean time 1.10 years vs. 1.05 years, *p* = 0.60). A majority of responders (80.9%) gave the reason to be routine dental examination. Toothache was reported as the reason by 146 (11.5%) respondents: 71 (10.9%) patients and 75 (12.1%) controls (*p* = 0.59). The composite variable was reported as the reason by 439 (32.7%) respondents: 217 (32.1%) patients and 222 (33.4%) controls (*p* = 0.64). No statistical difference was observed between patients and controls. Figure 2 illustrates the responses given by patients and controls, respectively.

### 3.2. Toothache and the Risk of MI

Table 2 presents the conditional logistic regression analysis with toothache as the independent variable and a first MI as the dependent variable, unadjusted and adjusted for confounders. Toothache was not associated with the risk of a first MI in the unadjusted or the adjusted analysis.

Table 3 presents the conditional logistic regression analysis with the composite variable as the independent variable and a first MI as the dependent variable, unadjusted and adjusted for confounders. The composite variable was not associated with the risk of a first MI in the unadjusted or the adjusted analysis.

## 4. Discussion

By analyzing survey data from the comprehensive PAROKRANK case–control study, the present paper assessed the self-reported reason for the latest visit to a dental office with the aim to investigate the prevalence of toothache prior to a first MI. Results did not disclose any difference between MI patients and controls in regard to toothache, and the conditional logistic regression analysis, unadjusted as well as adjusted for confounders, failed to associate toothache to the risk of MI. Likewise, a composite variable that accounted for either toothache, chewing problem, or other problems with the teeth, was not associated with the risk of a first MI.

### 4.1. Symptomatic Endodontic Inflammatory Disease and IHD

Besides shared risk factors (e.g., smoking, diabetes mellitus, and socioeconomic status), a biological link between endodontic inflammatory disease and IHD is feasible. This pathway is mainly rationalized through the spread of bacteria/endotoxins from the root canal to distant sites, or through production of pro-inflammatory cytokines that elevate a systemic inflammatory burden [11]. Epidemiologic studies have frequently reported positive associations between apical periodontitis and IHD [12,13,14,15,16], as well as to higher systemic levels of inflammatory markers [17]. In previous studies analyzing PAROKRANK data, specifically untreated apical periodontitis was found to associate to MI in younger individuals (<65 years) [8]. In comparison to root filled teeth with persistent apical periodontitis, untreated infected teeth associate more often with acute symptomatic episodes [18]. Based on the dissimilar intracanal microbial flora [19], as well as differences in the periapical inflammatory response [20,21], teeth with endodontic disease but different clinical characteristics potentially associate differently to systemic diseases, e.g., IHD. Whether symptomatic endodontic conditions in particular associates to systemic diseases has not been extensively studied.

In the present analysis, occurrence of toothache was assessed through survey responses, but the definite cause for the toothache is unknown as no clinical data or dental charts were reviewed. Adjustments were made for radiographically assessed endodontic variables, i.e., root filled teeth, periapical lesions, both in untreated and root filled teeth. However, the study design did not allow for assessment of other potentially painful conditions, such as marginal periodontitis or temporomandibular joint disorders. Although the present study found no association between toothache and MI, more studies on this topic should be commenced.

### 4.2. Heterotopic Craniofacial Pain of Cardiac Origin

Patients with craniofacial pain are a routine occurrence in dental practice [22]. Non-odontogenic craniofacial pain can be a major diagnostic challenge, and the fact that cardiac pain can manifest as craniofacial pain is still unfamiliar to many. During cardiac ischemia, nociceptive afferent fibers are activated and the concentrations of various pain transmitting mediators, such as bradykinin, thromboxane, and prostaglandins, are altered [23]. The pain perception is transmitted through sympathetic and vagal afferent fibers which converge with the trigeminal system in the spinal neurons. This explains the occurrence of craniofacial pain that essentially originates from the heart [24].

The scientific literature on craniofacial pain of cardiac origin is mostly limited to case reports [5,25,26,27,28], and a few cross-sectional [29,30] and prospective studies [31]. The prevalence of pain in the orofacial area during cardiac ischemic events amounts to between 18 to 38% of all patients [24,30,31] and is reported as the only symptom, with no accompanying chest pain, in approximately 6% [32]. Consequently, dental practitioners may play an important role in detecting such atypical symptoms of cardiac origin and, therefore, must be aware of the symptomatology in order to prevent unwarranted dental treatment and delay of medical care.

In the present analysis, roughly ten percent of patients reported toothache as the reason for their most recent visit to a dental office prior to the MI event. Whether some of these cases represent craniofacial pain of cardiac origin is unknown. Whilst craniofacial pain of cardiac origin is mostly reported as bilateral, dental pain is typically unilateral [26]. The most frequent craniofacial structures for cardiac pain are the throat and mandible, followed by the temporomandibular joint/ear region, and teeth [5]. The quality differs between pain of cardiac and dental origin, with the former more often described as tight, burning, and pressure and the latter as tingling, throbbing, and aching [33]. The present study design did not allow for detailed assessment of location or pain quality, which otherwise, together with information about additional cardiac symptoms, could have aided in distinguishing between toothache of odontogenic or cardiac origin.

The PAROKRANK study population consists primarily of male participants (81%) whose mean age was 62 ± 8 years. Previous studies on orofacial pain of cardiac origin have demonstrated a difference between women and men, with women experiencing craniofacial pain more frequently [29,31]. Additionally, whereas cardiac ischemia is diagnosed primarily in middle-aged and elderly patients, dental pain also frequently occurs in younger individuals [33]. MI patients and controls were matched for sex and age but the conditional logistic regression analysis revealed no association between toothache and MI.

Mean time since the last visit to a dental office was 1.1 years for MI patients and 1.05 years for controls. Prodromal angina, also called pre-infarction angina, is acute episodes of myocardial ischemia prior to an MI that can occur hours, weeks, or months, ahead of the acute event. Although not well known by either the public or clinicians, they can serve as early warnings signs of MI [28]. In the present study, adjusting for time since last visit to a dental office did not reveal an association between toothache and MI. Still, the possibility of prodromal angina to present itself as pain in the orofacial area should not be ignored.

### 4.3. Strengths and Limitations

The strength of the present study relates to the large and carefully characterized PAROKRANK population of more than 1600 individuals with clinical and radiographic information. Endodontic variables were assessed by three observers who underwent a thorough calibration process which increased the reliability in registrations of periapical disease [10]. Limitations relate to the assessment of toothache being solely based on survey data gathered in conjunction with the clinical examination of patients and controls. Recall bias is a risk when study participants are asked to recollect information in retrospect. Assessment of toothache was based on the response to a question that concerned the reason for the latest visit to a dental office. Questions regarding type, localization, and duration of pain could have allowed for more detailed interpretation of the results. Endodontic variables were assessed with panoramic radiography, a less sensitive method than other radiological techniques, especially for assessing the periapical status. Nevertheless, panoramic radiography has previously been considered suitable for epidemiologic studies [34].

## 5. Conclusions

Toothache did not predict a subsequent MI in the present analysis. It is yet unknown if oral conditions of certain characteristics, e.g., symptomatic ones, have a more prominent influence on systemic health outcomes than conditions with other characteristics, e.g., asymptomatic ones. The topic warrants further investigation. Also, physicians need to be mindful of atypical cardiac pain patterns and dentists should consider the possibility of rare causes, such as cardiac ischemia, to orofacial pain that fails to provide a diagnosis, in order to prevent misdirected dental treatment and delay of medical care.

## Figures and Tables

**Table 1:** Responses to time since, and reason for, last visit to a dental office by patients and controls.

Response Variables		Patients (*n* = 805)	Controls (*n* = 805)	*p*-Value	Difference Between Groups Mean (95% CI)
Time (years) since last visit to a dental office		1.10 (1.86)	1.05 (1.64)	0.60	0.05 (−0.13; 0.22)
		1 (0; 14)	1 (0; 14)		
		*n* = 772	*n* = 768		
Reason for last visit to a dental office					
	Routine dental examination				
	No	144 (19.7%)	129 (18.5%)		1.2 (−0.3; 5.4)
	Yes	588 (80.3%)	569 (81.5%)	0.61	−1.2 (−5.4; 3.0)
	Toothache				
	No	578 (89.1%)	547 (87.9%)		(−2.5; 4.8)
	Yes	71 (10.9%)	75 (12.1%)	0.59	−1.1 (−4.8; 2.5)
	Chewing problem				
	No	591 (92.6%)	571 (92.7%)		−0.1 (−3.1; 3.0)
	Yes	47 (7.4%)	45 (7.3%)	1.00	0.1 (−3.0; 3.1)
	Other problems with the teeth				
	No	517 (78.2%)	492 (76.4%)		1.8 (−2.9; 6.5)
	Yes	144 (21.8%)	152 (23.6%)	0.47	−1.8 (−6.5; 2.9)
	Improving the appearance of teeth				
	No	589 (92.3%)	561 (91.2%)		1.1 (−2.1; 4.3)
	Yes	49 (7.7%)	54 (8.8%)	0.54	−1.1 (−4.3; 2.1)
	Composite variable				
	No	460 (67.9%)	443 (66.6%)		1.3 (−6.5; 3.8)
	Yes	217 (32.1%)	222 (33.4%)	0.64	−1.3 (−6.5; 3.8)

For categorical variables *n* (%) is presented. For continuous variables Mean (SD)/Median (Min; Max)/(Bootstrapped (10,000 replicates) 95% CI for Mean/*n* is presented.

**Table 2:** Conditional logistic regression analysis for toothache and the risk of a first MI.

Variable	Odds Ratio	95% Confidence Interval
Unadjusted analysis		
Toothache	0.96	0.64–1.44
Adjusted analysis *		
Toothache	0.90	0.57–1.41
Time since last visit to a dental office	1.07	0.99–1.16
Root filled teeth	0.96	0.89–1.04
Teeth with periapical lesions	1.13	0.90–1.42
Root filled teeth with periapical lesions	0.97	0.71–1.32

* Adjustments are made for time since last visit to a dental office and endodontic variables (number of root filled teeth, number of teeth with periapical lesions and number of root filled teeth with periapical lesions).

**Table 3:** Conditional logistic regression analysis for the composite variable and the risk of a first MI.

Variable	Odds Ratio	95% Confidence Interval
Unadjusted analysis		
Composite variable	0.92	0.71–1.19
Adjusted analysis *		
Composite variable	0.85	0.65–1.13
Time since last visit to a dental office	1.07	0.99–1.16
Root filled teeth	0.98	0.91–1.05
Teeth with periapical lesions	1.12	0.90–1.40
Root filled teeth with periapical lesions	0.98	0.73– 1.32

* Adjustments are made for time since last visit to a dental office and endodontic variables (number of root filled teeth, number of teeth with periapical lesions, and number of root filled teeth with periapical lesions).

## Data Availability

No new data were created or analyzed in this study.

## Abbreviations

The following abbreviations are used in this manuscript:

IHD	Ischemic heart disease
MI	Myocardial infarction

## References

[1] GBD 2021 Causes of Death Collaborators (2024). Global burden of 288 causes of death and life expectancy decomposition in 204 countries and territories and 811 subnational locations, 1990–2021: A systematic analysis for the Global Burden of Disease Study 2021. Lancet.

[2] Ross R. (1999). Atherosclerosis—An inflammatory disease. N. Engl. J. Med..

[3] Jakovljevic A., Duncan H.F., Nagendrababu V., Jacimovic J., Milasin J., Dummer P.M.H. (2020). Association between cardiovascular diseases and apical periodontitis: An umbrella review. Int. Endod. J..

[4] Farges J.C., Alliot-Licht B., Renard E., Ducret M., Gaudin A., Smith A.J., Cooper P.R. (2015). Dental Pulp Defence and Repair Mechanisms in Dental Caries. Mediat. Inflamm..

[5] Choi E., Lee Y.H., Park H.K. (2023). Orofacial Pain with Cardiac Origin of Coronary Artery Disease: A Case Report and Literature Review. Case Rep. Dent..

[6] Chan W.K., Leung K.F., Lee Y.F., Hung C.S., Kung N.S., Lau F.L. (1998). Undiagnosed acute myocardial infarction in the accident and emergency department: Reasons and implications. Eur. J. Emerg. Med..

[7] Ryden L., Buhlin K., Ekstrand E., de Faire U., Gustafsson A., Holmer J., Kjellstrom B., Lindahl B., Norhammar A., Nygren A. (2016). Periodontitis Increases the Risk of a First Myocardial Infarction: A Report From the PAROKRANK Study. Circulation.

[8] Sebring D., Buhlin K., Norhammar A., Ryden L., Jonasson P., EndoReCo, Lund H., Kvist T. (2022). Endodontic inflammatory disease: A risk indicator for a first myocardial infarction. Int. Endod. J..

[9] Thygesen K., Alpert J.S., Jaffe A.S., Simoons M.L., Chaitman B.R., White H.D., Writing Group on the Joint ESC/ACCF/AHA/WHF Task Force for the Universal Definition of Myocardial Infarction (2012). Third universal definition of myocardial infarction. Circulation.

[10] Sebring D., Kvist T., Buhlin K., Jonasson P., EndoReCo, Lund H. (2021). Calibration improves observer reliability in detecting periapical pathology on panoramic radiographs. Acta Odontol. Scand..

[11] Segura-Egea J.J., Martin-Gonzalez J., Castellanos-Cosano L. (2015). Endodontic medicine: Connections between apical periodontitis and systemic diseases. Int. Endod. J..

[12] Willershausen B., Kasaj A., Willershausen I., Zahorka D., Briseno B., Blettner M., Genth-Zotz S., Munzel T. (2009). Association between chronic dental infection and acute myocardial infarction. J. Endod..

[13] Willershausen I., Weyer V., Peter M., Weichert C., Kasaj A., Munzel T., Willershausen B. (2014). Association between chronic periodontal and apical inflammation and acute myocardial infarction. Odontology.

[14] An G.K., Morse D.E., Kunin M., Goldberger R.S., Psoter W.J. (2016). Association of Radiographically Diagnosed Apical Periodontitis and Cardiovascular Disease: A Hospital Records-based Study. J. Endod..

[15] Virtanen E., Nurmi T., Soder P.O., Airila-Mansson S., Soder B., Meurman J.H. (2017). Apical periodontitis associates with cardiovascular diseases: A cross-sectional study from Sweden. BMC Oral Health.

[16] Leao T.S.S., Tomasi G.H., Conzatti L.P., Marrone L.C.P., Reynolds M.A., Gomes M.S. (2022). Oral Inflammatory Burden and Carotid Atherosclerosis Among Stroke Patients. J. Endod..

[17] Gomes M.S., Blattner T.C., Sant’Ana Filho M., Grecca F.S., Hugo F.N., Fouad A.F., Reynolds M.A. (2013). Can apical periodontitis modify systemic levels of inflammatory markers? A systematic review and meta-analysis. J. Endod..

[18] Yu V.S., Messer H.H., Yee R., Shen L. (2012). Incidence and impact of painful exacerbations in a cohort with post-treatment persistent endodontic lesions. J. Endod..

[19] Molander A., Reit C., Dahlen G., Kvist T. (1998). Microbiological status of root-filled teeth with apical periodontitis. Int. Endod. J..

[20] Chen S., Lei H., Luo Y., Jiang S., Zhang M., Lv H., Cai Z., Huang X. (2019). Micro-CT analysis of chronic apical periodontitis induced by several specific pathogens. Int. Endod. J..

[21] Chow A.T., Quah S.Y., Bergenholtz G., Lim K.C., Yu V.S.H., Tan K.S. (2019). Bacterial species associated with persistent apical periodontitis exert differential effects on osteogenic differentiation. Int. Endod. J..

[22] McCarthy P.J., McClanahan S., Hodges J., Bowles W.R. (2010). Frequency of localization of the painful tooth by patients presenting for an endodontic emergency. J. Endod..

[23] Kreiner M., Okeson J.P. (1999). Toothache of cardiac origin. J. Orofac. Pain..

[24] Kreiner M., Okeson J., Tanco V., Waldenstrom A., Isberg A. (2020). Orofacial Pain and Toothache as the Sole Symptom of an Acute Myocardial Infarction Entails a Major Risk of Misdiagnosis and Death. J. Oral Facial Pain. Headache.

[25] Turner M.J., McMillan K.G., Gibbons A.J. (2013). Angina presenting as orofacial pain: A case report. Oral Surg. Oral Med. Oral Pathol. Oral Radiol..

[26] Lopez-Lopez J., Garcia-Vicente L., Jane-Salas E., Estrugo-Devesa A., Chimenos-Kustner E., Roca-Elias J. (2012). Orofacial pain of cardiac origin: Review literature and clinical cases. Med. Oral Patol. Oral Cir. Bucal.

[27] Adachi M., Hayashi M., Segawa T., Yamaki T., Muramatsu Y., Sumitomo S. (2017). Orofacial Pain Associated with Vasospastic Angina: A Case Report. J. Oral Facial Pain. Headache.

[28] Fazlyab M., Esnaashari E., Saleh M., Shakerian F., Moayed D.A., Asgary S. (2015). Craniofacial Pain as the Sole Sign of Prodromal Angina and Acute Coronary Syndrome: A Review and Report of a Rare Case. Iran. Endod. J..

[29] Kreiner M., Alvarez R., Waldenstrom A., Michelis V., Muniz R., Isberg A. (2014). Craniofacial pain of cardiac origin is associated with inferior wall ischemia. J. Oral Facial Pain. Headache.

[30] Bakhshi M., Rezaei R., Baharvand M., Bakhtiari S. (2017). Frequency of craniofacial pain in patients with ischemic heart disease. J. Clin. Exp. Dent..

[31] Danesh-Sani S.H., Danesh-Sani S.A., Zia R., Faghihi S. (2012). Incidence of craniofacial pain of cardiac origin: Results from a prospective multicentre study. Aust. Dent. J..

[32] Jalali N., Vilke G.M., Korenevsky M., Castillo E.M., Wilson M.P. (2014). The tooth, the whole tooth, and nothing but the tooth: Can dental pain ever be the sole presenting symptom of a myocardial infarction? A systematic review. J. Emerg. Med..

[33] Kreiner M., Falace D., Michelis V., Okeson J.P., Isberg A. (2010). Quality difference in craniofacial pain of cardiac vs. dental origin. J. Dent. Res..

[34] Molander B., Ahlqwist M., Grondahl H.G., Hollender L. (1993). Comparison of panoramic and intraoral radiography for the diagnosis of caries and periapical pathology. Dentomaxillofac. Radiol..

